# Integrated Machine Learning and Bioinformatic Analyses Constructed a Novel Stemness-Related Classifier to Predict Prognosis and Immunotherapy Responses for Hepatocellular Carcinoma Patients

**DOI:** 10.7150/ijbs.66913

**Published:** 2022-01-01

**Authors:** Dongjie Chen, Jixing Liu, Longjun Zang, Tijun Xiao, Xianlin Zhang, Zheng Li, Hongwei Zhu, Wenzhe Gao, Xiao Yu

**Affiliations:** 1 Department of Hepatopancreatobiliary Surgery, The Third Xiangya Hospital, Central South University, Changsha, Hunan, P.R. China.; 2 Department of Nephrology, Institute of Nephrology, 2nd Affiliated Hospital of Hainan Medical University, Haikou, Hainan, P.R. China.; 3 Department of General Surgery, Shaoyang University Affiliated Second Hospital, Shaoyang University, Shaoyang, Hunan, P.R. China.; 4 Department of General Surgery, Affiliated Renhe Hospital of China Three Gorges University, Yichang, Hubei, P.R. China.

**Keywords:** Cancer stem cell, Immunotherapy, Machine learning, Hepatocellular carcinoma

## Abstract

Immunotherapy has made great progress in hepatocellular carcinoma (HCC), yet there is still a lack of biomarkers for predicting response to it. Cancer stem cells (CSCs) are the primary cause of the tumorigenesis, metastasis, and multi-drug resistance of HCC. This study aimed to propose a novel CSCs-related cluster of HCC to predict patients' response to immunotherapy. Based on RNA-seq datasets from The Cancer Genome Atlas (TCGA) and Progenitor Cell Biology Consortium (PCBC), one-class logistic regression (OCLR) algorithm was applied to compute the stemness index (mRNAsi) of HCC patients. Unsupervised consensus clustering was performed to categorize HCC patients into two stemness subtypes which further proved to be a predictor of tumor immune microenvironment (TIME) status, immunogenomic expressions and sensitivity to neoadjuvant therapies. Finally, four machine learning algorithms (LASSO, RF, SVM-RFE and XGboost) were applied to distinguish different stemness subtypes. Thus, a five-hub-gene based classifier was constructed in TCGA and ICGC HCC datasets to predict patients' stemness subtype in a more convenient and applicable way, and this novel stemness-based classification system could facilitate the prognostic prediction and guide clinical strategies of immunotherapy and targeted therapy in HCC.

## Introduction

Liver cancer remains one of the most common and lethal malignancies around the world. Although the incidence has been effectively controlled in the past decades, it is estimated that liver cancer will still cause 905,677 new cases and 830,180 new deaths worldwide in 2021 1. Hepatocellular carcinoma (HCC) is the main histological subtype of liver cancer, accounting for about 90% of primary liver cancer cases. HCC is still one of the worst prognostic malignancies, with a 5-year survival rate of only about 20% 2. Surgical interventions are still regarded as the backbone for the treatment of HCC patients; however, strict surgical indications have determined that a majority of HCC patients can only take other conservative treatments such as transarterial chemoembolization (TACE), local ablation and systemic therapies 3. Moreover, it is worth mentioning that modern targeted therapy has made great progress in HCC treatment. A combining regimen of atezolizumab (anti-PDL1 antibody) and bevacizumab (anti-VEGF antibody), compared with the classic first-line chemotherapy based on sorafenib, has shown better ability to improve the prognosis of patients 4. However, side effects, high economic burden and survival benefits are still not ideal enough to remind us that research on the molecular pathogenesis of HCC still has a long way to go.

Cancer stem cells (CSCs) refer to a bunch of tumor cells that possess self-renewal and differentiation potential. Since first identified in the 1990s in leukemia marked by CD34+/CD38-[5], CSCs have been subsequently found in many nonsolid and solid tumors. These cells are believed to initiate tumorigenesis and cause many other malignant biological behaviors, including epithelial-mesenchymal transition (EMT, leading to local invasion and distant metastasis), cancer recurrence and drug resistance, et al. Not surprisingly, CSCs also play a pivotal part in the carcinogenesis and development of HCC 6. However, traditional experimental methods for sorting CSCs, such as fluorescence-activated cell sorting (FACS) and magnetic-activated cell sorting (MACS) 7, remain controversial for their low ratio of enriched CSCs. Thus, research on CSCs also requires new strategies for further development.

Immunotherapy is the current research hotspot in tumor therapeutics, bringing new hope to patients with advanced cancers. As mentioned above, immune checkpoint inhibitor (ICI) against PD-1/PD-L1 has been proven active, tolerable and clinically beneficial in advanced HCC patients and has been clinically applied as a first-line therapy worldwide. HCC can also be classified as a so-called “cold tumor” for its low tumor mutational burden (TMB) 8, enrichment of exhausted or dysfunctional tumor-infiltrating lymphocytes (TILs) 9 and dampened innate immune response 10. Mechanism research has shown that the atezolizumab plus bevacizumab combination can synergistically produce the following effects: 1) inhibiting angiogenesis; 2) reducing the infiltration of myeloid-derived suppressor cells (MDSCs) and adjusting the ratio of M1/M2 tumor-associated macrophages (TAMs); 3) promoting the infiltration and functional recovery of CD8+ T cells (CTLs) 11. These may be the reason why anti-PDL1 immunotherapy has achieved favorable results in HCC. Nevertheless, there are still plenty of unanswered questions about immunotherapy in HCC. Firstly, it is still necessary to select drugs that would make the most of the immune-checkpoint blockade (ICB)-anti-angiogenic effect through further clinical trials. Secondly, the curative effect of other promising forms of immunotherapy, including additional ICBs, adoptive T cell transfer and vaccinations, have not been adequately studied in HCC. Last but not least, there is almost a complete lack of biomarkers to predict the response or to guide the choice and development of ICBs in HCC patients.

Recent studies have shown that there is a complex cross-link between the intrinsic stem cell nature of CSCs and the extrinsic immunosuppressive TME. On the one hand, signals like cytokines and growth factors from TME can activate stem cell signals such as EMT, Wnt, JAK/STAT and NFκB, enhancing tumor progression, metastasis, relapse and therapeutic resistance. On the other hand, these intrinsic signals in CSC can in turn induce TME remodeling, like angiogenesis, collagen remodeling and PD-1/PD-L1 related immune escape 12. These results prompt that a deeper exploration of the relationship between CSC markers and TME may point us to new strategies that can improve the fate of HCC patients receiving immunotherapy.

In this study, stemness index (mRNAsi) for HCC patients were evaluated based on transcriptome data. Then, based on the mRNAsi scores, patients were categorized into two stemness subtypes with distinguished prognostic status, clinical features functional annotations and tumor mutation burdens. Then, integrated bioinformatic analysis was applied to distinguish tumor microenvironment status, immunogenomic patterns and sensitivity to targeted chemotherapies. Furthermore, four machine learning algorithms were applied to construct a stemness subtype classifier based on the expression of several hub genes. This classifier provided a clinically applicable method for identifying patients who are more probably to have a sensitive response to immunotherapy based on stemness features.

## Materials and Methods

### Data Processing

The RNA-Seq data of pluripotent stem cells (PSCs) were obtained from the Progenitor Cell Biology Consortium (PCBC) database (https://www.synapse.org) via the 'synapser' package in the R program. The gene expression matrix of HCC patients were extracted from The Cancer Genome Atlas (TCGA-LIHC) database ((https://portal.gdc.cancer.gov/projects/TCGA-LIHC) and the International Cancer Genome Consortium (ICGC) database (https://dcc.icgc.org/releases/current/Projects/LIRI-JP) separately. After ruling out specimens without complete clinicopathological information, we extracted 365 patients from the TCGA and 231 patients from the ICGC dataset as external validation. For enabling the comparability of two different databases and minimizing the batch effect, FPKM (fragments per kilobase of transcript per million fragments mapped) data of RNA-Seq were log2 transformed and then normalized via the R package 'caret'. Additionally, the somatic mutation landscape and copy number variation (CNV) data of HCC patients were also downloaded from the TCGA website, and visualized by R package “maftools” and “RCircos”.

### Calculation of the stemness index (mRNAsi)

Derived from the mean-centered RNA-Seq data of PSCs in the PCBC database (syn2701943), the stemness signature was identified via the one-class logistic regression (OCLR) machine learning algorithm 13, which was also verified by leave-one-out cross-validation. Next, the Spearman correlation analysis was conducted between the normalized expression matrix of HCC samples and the stemness hallmarks. Eventually, the stemness index was identified by scaling the Spearman correlation coefficients to be between 0 and 1. The higher the mRNAsi, the greater the tumor dedifferentiation and higher stemness 14.

### Differential gene expression analysis

Based on the median value of the mRNAsi calculated mentioned above, HCC patients in TCGA dataset were divided into high- and low- mRNAsi subgroups. The 'DESeq2' package was utilized to identify differential genes (DEGs) among high- and low- mRNAsi subgroup 15. False discovery rate (FDR) method was applied to correct the results at *P*<0.01 level. The screening criteria was |log2 fold change (FC)| >2 and (FDR) <0.01. Furthermore, Gene Ontology (GO) and Kyoto encyclopedia of genes and genomes (KEGG) enrichment analyses were conducted utilizing 'clusterProfiler' package in R 16.

### Determination of the stemness-based classification

Consensus clustering, an unsupervised class discovery method, was applied to identify a novel stemness-based classification via the 'ConsensusClusterPlus' R package 17. This clustering process was repeated for 1000 repetitions by subsampling 80% of items, partitioning each subsample into several groups by k-means algorithm. The consensus matrix (CM) plot and cumulative distribution function (CDF) plot were visualized to find the optimal number of clusters. Then, Kaplan-Meier (K-M) curve was conducted to appraise the overall survival (OS) of different stemness subtypes.

### Identification of the tumor immune infiltrating features of HCC patients

CIBERSORT, an analytical tool, was utilized to import unnormalized RNA-Seq data of HCC patients and provide an evaluation of the relative abundance of 22 immune-related cell types in a mixed cell population 18. In addition, Estimation of STromal and Immune cells in MAlignant Tumor tissues using Expression data (ESTIMATE) algorithm was implemented to predict the presence of infiltrating stromal/immune cells based on the expression profiles of HCC patients 19. ESTIMATE algorithm generates three scores: stromal score (that seizes the abundance of stroma), immune score (that symbolizes the infiltration of immune cells) and estimate score (that represents tumor purity). Using the R package 'GSVA', we also performed a single sample Gene Set Enrichment Analysis (ssGSEA) to compute the enrichment scores of each immune-related term which was quantified by 29 immune signatures^20^, ^21^. Then, based on the ssGSEA scores of HCC patients, we performed an unsupervised hierarchical clustering method to distribute HCC patients into different immune subtypes via R package “pvclust”.

### Gene set variation analysis (GSVA)

GSVA was conducted to assess the variation of pathway activity in different stemness subtypes via 'GSVA' package in an unsupervised manner. The gene set 'h.all.v7.4.symbols.gmt' and 'c2.cp.kegg.v7.4.symbols.gmt' were downloaded from MSigDB database and selected as the background gene set. R package 'limma' was utilized to perform differential analysis of KEGG and HALLMARK pathways between two stemness subtypes. The criteria of the significant enrichment pathways are as follows: FDR<0.05 and |log2 fold change (FC)| >0.1.

### Identification of candidate small molecules

The Connectivity Map (CMap, https://clue.io/) is a web-based database that uses cellular responses to perturbation to find relationships between diseases, genes, and therapeutics 22. DEGs between different mRNAsi subgroups were queried to be compared for similarity to all perturbational signatures in the database, and compounds with negatively enrichment scores were selected to predict their mode of action (MoA).

### Prediction of immunotherapy and chemotherapy

The expression levels of several immune checkpoints (PD1, PD-L1, PD-L2, CTLA4, CD80, CD86) were screened to evaluate the sensitivity of immunotherapy among two stemness subtypes. Also, based on the Cancer Cell Line Encyclopedia (CCLE) database, the 'pRRophetic' package was used to predict the chemotherapeutic response to erlotinib quantified by the half-maximal inhibitory concentration (IC50) of each HCC patient 23, 24. IC50 is a measure of the potency of a compound in suppressing a distinct biological or biochemical function in HCC. Generally speaking, the less the IC50, the stronger the sensitivity to specific compound. Finally, a ridge regression model was fit to expression data of HCC patients and the predictive power was measured by 5-fold cross-validation.

### Development and verification of the stemness-based classifier via multiple machine learning algorithms

A total of 365 HCC patients with complete clinical information were categorized into training (n=255) and testing (n=110) set at an equal ratio of 7:3. After removing the attributes with an absolute correlation of 0.75 or higher, the expression of 56 DEGs among the high- and low- mRNAsi subgroup were enrolled. Aimed at accurately predicting the status of stemness subtype, least absolute shrinkage and selection operator (LASSO) regression, support vector machine recursive feature elimination (SVM-RFE), Random Forest and Boruta (RFB), and extreme gradient boosting (XGBoost) analyses were applied to rank features by importance via 'glmnet', 'rms', 'sigFeature', 'e1071', 'randomForest', 'Boruta', 'XGBoost' R packages 25-28. Genes converged by these four machine learning methods for feature selection were confirmed as the most relevant and feasible characteristics of the stemness subtype. Furthermore, multivariate logistic regression was implemented to construct the stemness-based classifier 29. The formula of the classifier was as follows:



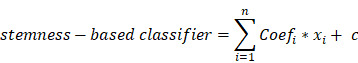



where 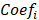 represents the coefficients, 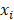 refers to the normalized expression value of converged genes,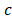 means the intercept. Then, the classifier was normalized to the range of 0 to 1. The capacity of the prediction based on the multivariate logistic model was depicted by the time-dependent receiver operating characteristic (ROC) curve which in turn determine the optimal cutoff value of the classifier, with the evaluation of the area under the ROC curve (AUC) using the 'pROC' package. Ultimately, the stemness-based classifier was verified in the testing set and validation set by the same token.

### Statistical analysis

Spearman correlation test was utilized to estimate the relationships between two variables that are not linearly related. The student's t-test was used to compare the normally distributed data. Chi-square test was utilized to compare the categorical and pairwise features of different subgroup. Wilcoxon test was applied to compare ordinal and non-normally distributed data between different subgroups. Kruskal-Wallis test was used for one independent variable with two or more levels and an ordinal dependent variable. K-M analysis was used to measure the fraction of HCC patients living for a certain amount of time and the log-rank test was performed to evaluate the significance of differences. The statistical analyses were conducted in this study by using the R program (Version 4.1.0). A two-tailed *p*-value of less than 0.05 was deemed to be statistically significant unless specifically stated.

## Results

### Correlation between mRNAsi and clinicopathological characteristics of HCC patients

**Figure 1:**
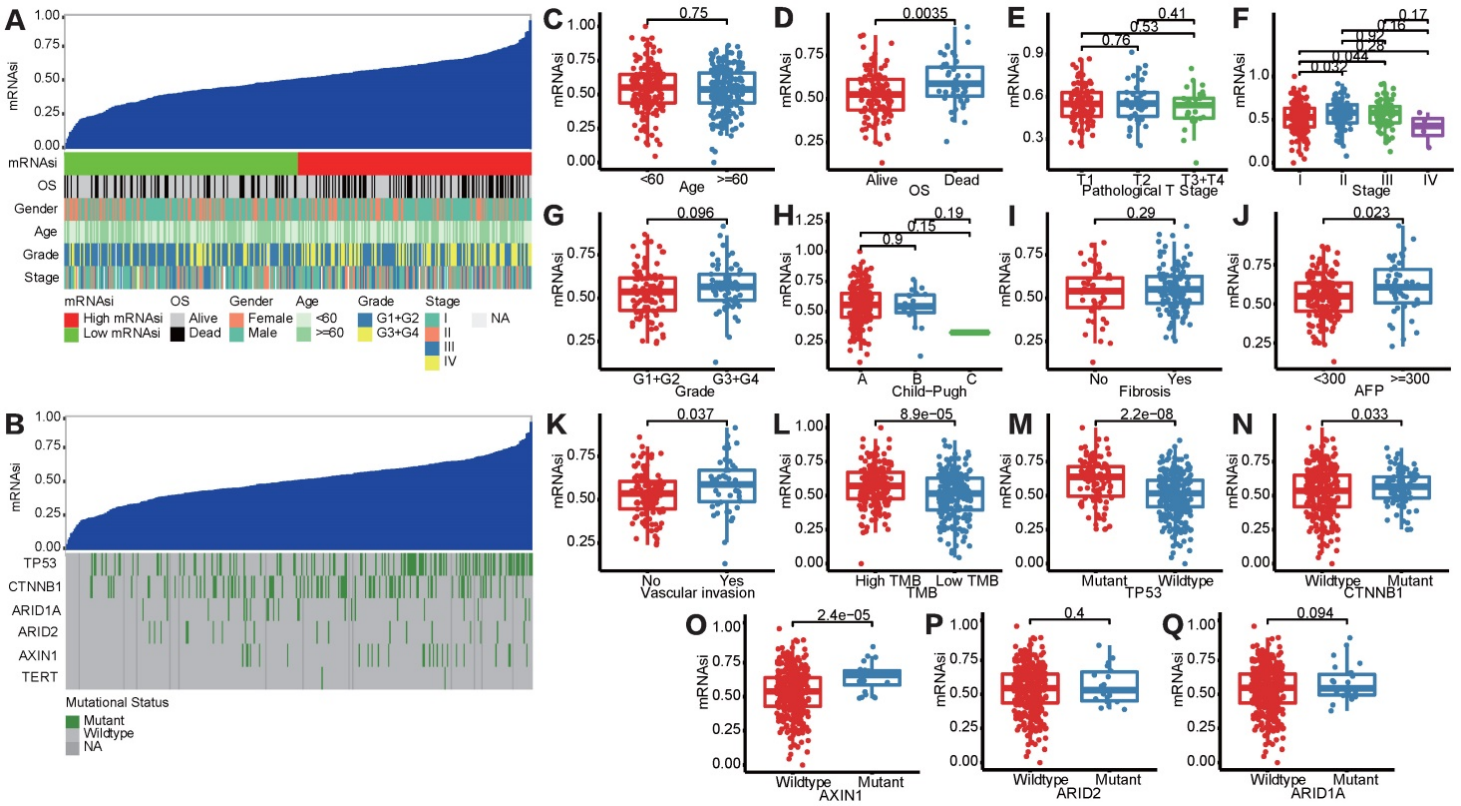
** Correlation between mRNAsi scores and clinical features in HCC patients. (A)** An overview of the association between mRNAsi and clinicalpathological characteristics of HCC patients. **(B)** An overview of the association between mRNAsi and somatic mutations of the most popular biomarkers of HCC. **(C-Q)** Boxplots of mRNAsi scores for HCC patients stratified by clinical features and mutational status of HCC biomarkers.

Figure S1 showed the workflow chart of the whole study. Based on the OCLR algorithm mentioned in the Materials and Methods part, we first calculated the stemness index (mRNAsi) for each patient in TCGA-LIHC cohort using RNA-seq data. Then, we ranked every patient from low to high according to mRNAsi scores and detected the relationship between this index and clinicopathological features (Figure 1A). HCC patients with survival status “Deceased” (Figure 1D), AFP ≥300 ng/mL (Figure 1J) and vascular invasion happened (Figure 1K) had a significantly higher mRNAsi score compared to the corresponding control group. In addition, patients diagnosed with clinical stage II showed a significantly higher mRNAsi score than stage I patients, but no trend was revealed regarding patients with more advanced clinical stages (Figure 1F). Moreover, we also integrated data of somatic mutations that were commonly happened in HCC (Figure 1B), it turned that patients with higher TMB tended to get higher mRNAsi as well (Figure 1L). Besides, regarding the most common mutation events of HCC, mRNAsi was significantly higher than wild-type samples when TP53 (Figure 1M), CTNNB1 (Figure 1N) and AXIN1 (Figure 1O) mutation happened.

### Correlation between mRNAsi and TIME patterns

**Figure 2:**
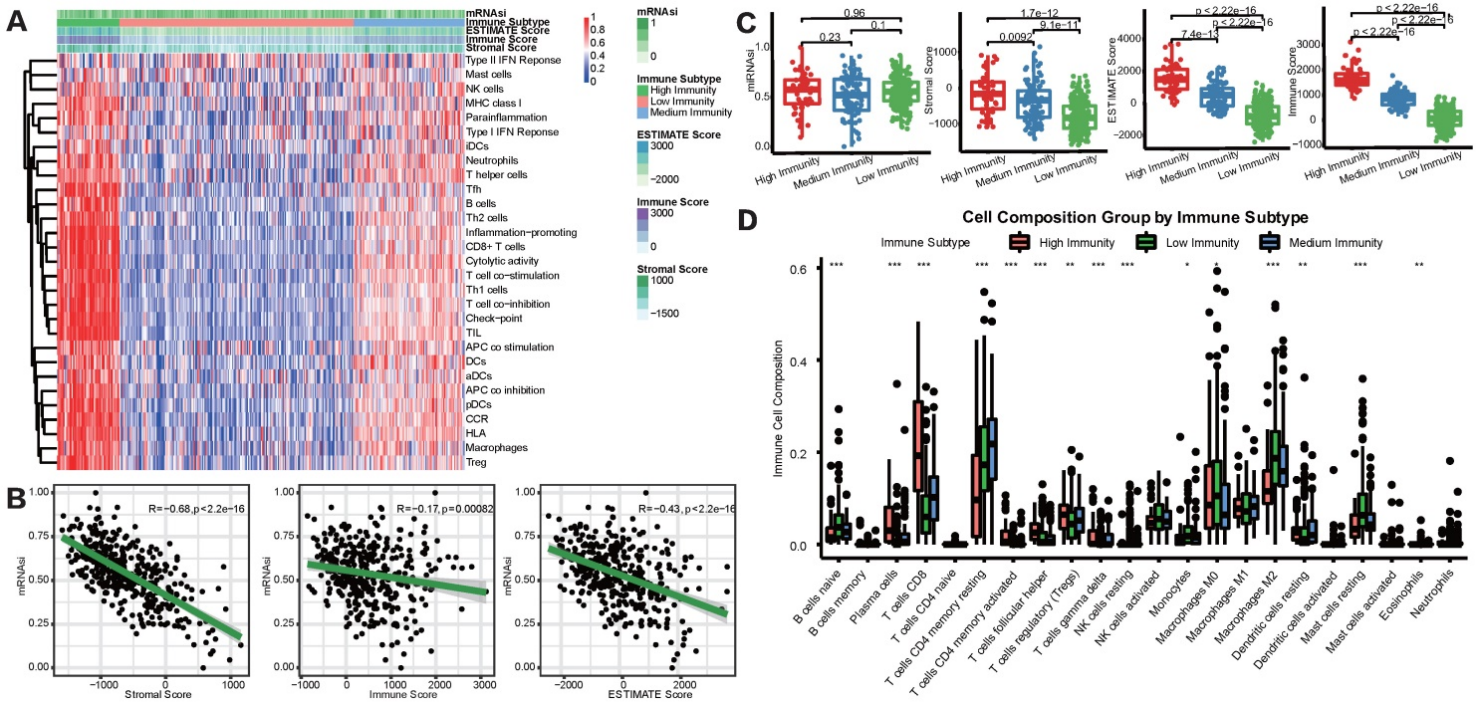
** mRNAsi scores were correlated with different TIME patterns of HCC patients. (A)** HCC patients were categorized into 3 immune subgroups (high, medium and low immunity) based on ssGSEA immune signatures.** (B)** Correlation analysis between mRNAsi and the stromal score, immune score and ESTIMATE score by ESTIMATE algorithm. **(C)** Comparisons of mRNAsi, the infiltration level of stromal and immune cells and the ESTIMATE score in different immune subtypes by boxplots. **(D)** Comparisons of the abundances of 22 immune cells in three immune subtypes by CIBERSORT (*p < 0.05; **p < 0.01; ***p < 0.001; ****p < 0.0001; ns, *p* > 0.05).

To overview the status of immune infiltration for TCGA-LIHC cohort, ssGSEA was applied to quantify the enrichment of 29 immune hallmarks 21. Then unsupervised consensus clustering algorithm was utilized to cluster HCC patients into 3 immune subgroups. As shown in Figure 2A, the high-immunity subgroup, characterized by enrichment of nearly all of the 29 immune signatures, represented 15.9% of all HCC patients, while medium-immunity and low-immunity subgroups contained 23.8% and 60.3%, respectively. We defined these subgroups as “hot tumors”, “altered tumors” and “cold tumors” according to their different immune enrichment scores calculated by ssGSEA. Afterward, TIME status was determined by ESTIMATE and CIBERSORT algorithms. We found that mRNAsi was negatively correlated with the Immune, Stromal, and ESTIMATE scores (Figure 2B), prompting a significant association between higher degree of stemness and immunosuppression in HCC. In addition, we explored the relationship between the three immune clusters based on ssGSEA algorithm and mRNAsi score through boxplots (Figure 2C). Although there was none of the significant result discovered, we did find that the high-immunity subgroup, with the highest immune and stromal scores, tended to show a relatively higher average mRNAsi score. Besides, we also detected the infiltration abundance of 22 immune cells by CIBERSORT algorithms (Figure 2D), results showed that the differences in immune cell infiltration were significant between the 3 subgroups. For example, CD8+ T cells, which were effector killer T cells in tumor immune response, were significantly enriched in the high-immunity subgroup, while the immunosuppressive M2 macrophages were mainly enriched in the low-immunity subgroup.

### Identification of stemness subtypes based on the differential expression analysis of mRNAsi

**Figure 3:**
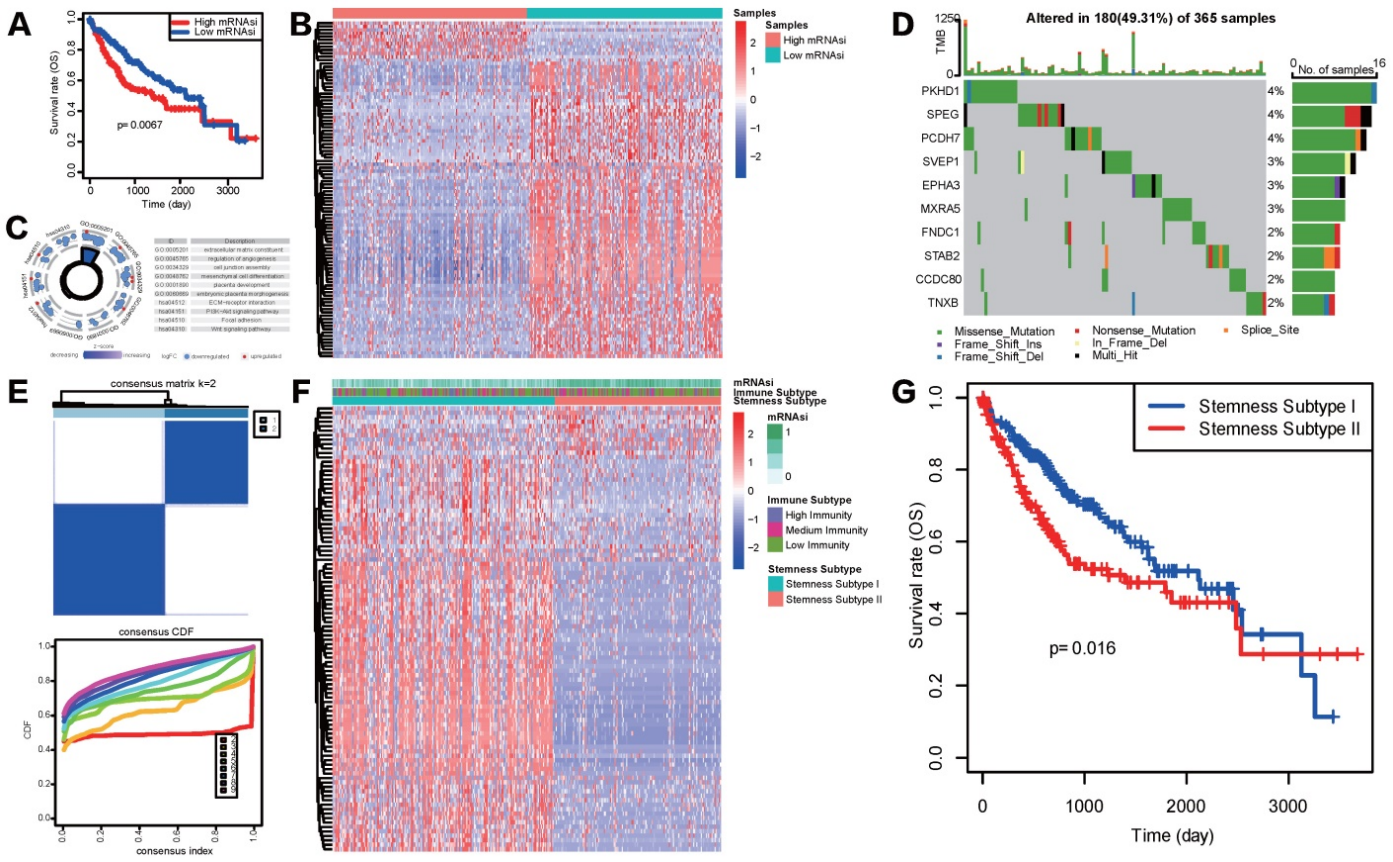
** Construction of stemness subtypes with distinct functional annotation and survival outcomes. (A)** K-M survival analyses indicated significantly worse OS in the high mRNAsi groups. **(B)** Heatmap showed the expression levels of 100 DEGs between high and low mRNAsi subgroups. **(C)** GO and KEGG functional enrichment analyses of DEGs. **(D)** Waterfall plot showed the mutational frequency of 10 most frequently mutated DEGs. **(E)** Results for consensus clustering based on the expression patterns of 100 DEGs. **(F)** The heatmap of the expression patterns of 100 DEGs and the integrated results of mRNAsi subgroups, immunity subgroups and stemness clusters. **(G)** K-M survival analysis showed the different survival status between two stemness subtypes.

Considering the above outcomes showed that mRNAsi was correlated with clinicopathological characteristics and tumor immune microenvironment patterns of HCC , we divided patients in TCGA-LIHC cohort into mRNAsi-high and mRNAsi-low subgroups based on the median score of mRNAsi and conducted K-M survival analysis to see if mRNAsi was significantly related to patients' overall survival (OS) in HCC. Result showed that patients with high mRNAsi score presented a significantly poorer OS status (Figure 3A). To further explore the underlying mechanism of these two mRNAsi subgroups mentioned above and characterize the functional role of mRNAsi phenotype-based system, we identified a novel stemness-based classification of HCC patients via multiple analyses. First, we performed differential expression analysis between mRNAsi-high and mRNAsi-low subgroups. A total of mRNAsi phenotype-based 100 DEGs were screened, including 12 upregulated and 88 downregulated genes in mRNAsi-high group (Figure 3B). GO and KEGG enrichment analysis revealed that these 100 mRNAsi phenotype-based DEGs were mainly enriched in pathways related to TME, such as extracellular matrix structural constituent, cell junction assembly, regulation of angiogenesis, mesenchymal cell differentiation, ECM receptor interaction and focal adhesion; some classical cancer pathways were emerged as well, like PI3K-AKT signaling and WNT signaling pathways (Figure 3C). Further waterfall plot for mutation analysis showed that 19 of 100 mRNAsi phenotype-based DEGs had a mutation frequency > 1%, all of the 19 genes were down-regulated in mRNAsi-high group and missense mutations were the most common mutation type (Figure 3D).

Given the heterogeneity of stemness characteristics, based on the expression patterns of the 100 mRNAsi phenotype-based DEGs, we utilized unsupervised consensus clustering to construct a specific stemness-based classification of HCC patients. As shown in Figure 3E and 3F, patients were categorized into 2 subgroups, defined as Stemness Subtype I (209 cases, 57.26%) and Stemness Subtype II (156 cases, 42.74%). As is shown in Figure 3F, stemness subtypes II tended to have mRNAsi scores. Besides, K-M curve illustrated that HCC patients in Stemness Subtype II possessed a markedly worse prognosis compared with cases in Stemness Subtype I (Figure 3G), preliminary indicating that this classifier was a prognostic indicator and had potentials for follow-up research.

### Stemness subtypes had distinct functional annotations, clinical features and CNV/mutation patterns

**Figure 4:**
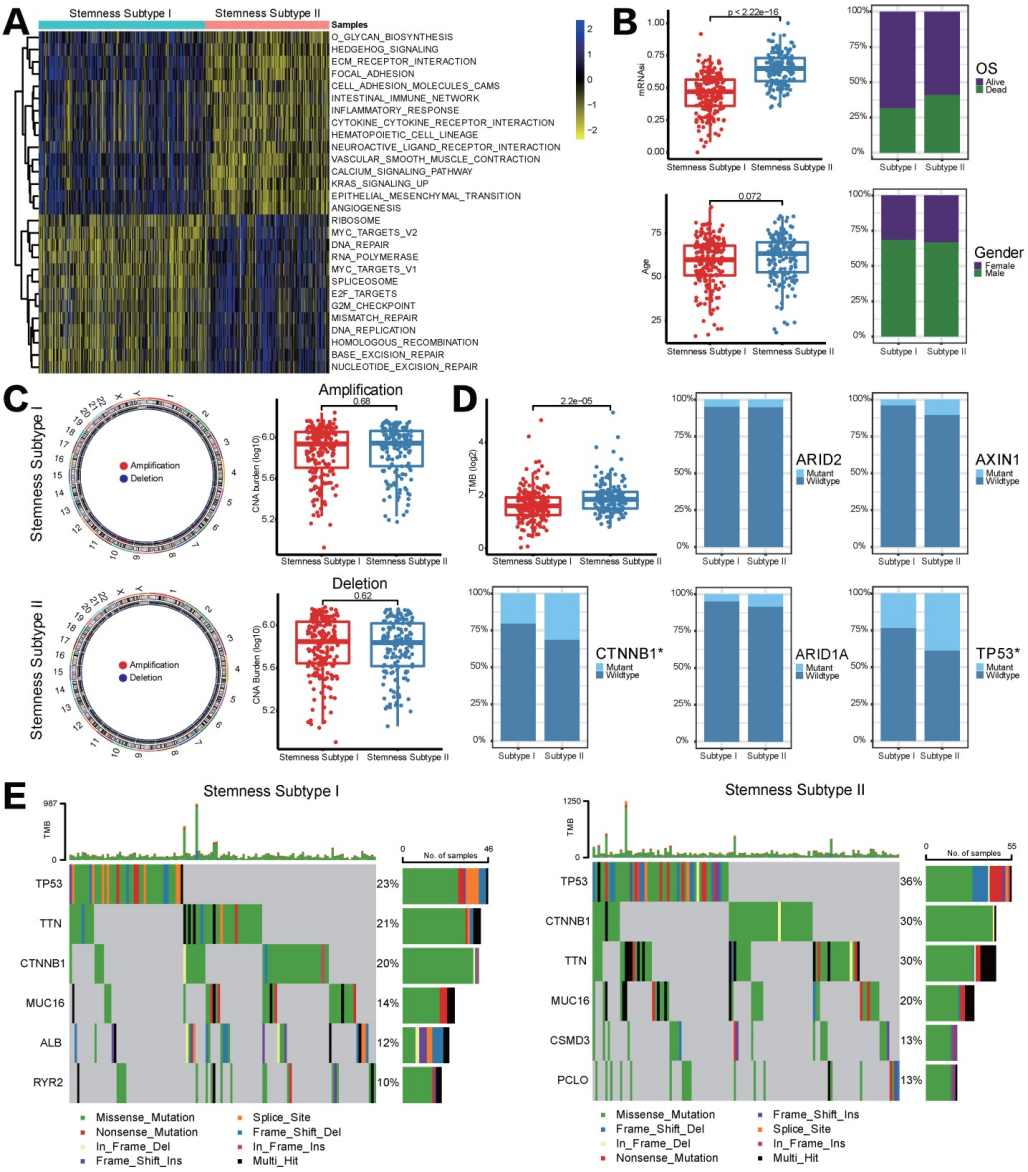
** Different functional annotations, clinicalpathological characteristics, CNV and TMB between two stemness subtypes. (A)** GSVA heatmap demonstrated the enrichment scores of 28 differentially enriched molecular pathways between two stemness subtypes.** (B)** Comparisons of age, mRNAsi, OS status and gender between two subtypes. **(C)** CNV frequencies for different stemness subtypes.** (D)** TMB difference and mutation status between two stemness subtypes.** (E)** Waterfall plots showed the most frequently mutated genes in two subtypes, respectively.

GSVA pathway analysis was applied to determine the underlying molecular mechanism that could distinguish stemness subtypes in HCC. A total of 28 pathways were differentially enriched between stemness subtypes I and II, including 13 pathways that significantly associated with stemness subtypes I and another 15 pathways markedly were enriched in stemness subtypes II. In detail, pathways related to cell cycle and DNA repair were the main enrichen in stemness subtypes I, such as MYC, G2/M checkpoint, etc. Nevertheless, EMT, ECM and immune-related pathways were mainly enriched in stemness subtypes II (Figure 4A). Afterward, we compared differences in clinical features between two stemness subtypes. Figure 4B showed that patients in stemness subtype II proved to be older, to be female and to have more “deceased” OS status, although it is not significant; besides, mRNAsi score in stemness subtypes II was significantly higher than that in subtypes I, indicating patients with stemness subtype II had a higher degree of neoplastic stemness, which implied stronger tendency to self-renewal and differential potency of HCC cells, these results may account for their worse OS.

As mentioned in Introduction section, genomic alterations including mutations and CNVs could play a key role in the regulation of tumor immunity. Given that we performed CNV and somatic mutation analysis in different stemness subtypes. However, the frequency of events of copy number amplifications or deletions did not differ significantly between stemness subtypes I and II (Figure 4C). Somatic mutation analysis revealed that TMB was significantly higher in patients in stemness subtypes I, and the mutation status of CTNNB1 and TP53 among two stemness subtypes was statistically significant (CTNNB1, *p*=0.03707, TP53, *p*=0.009588, Figure 4D). Furthermore, waterfall plots depicted high-frequency mutations in different genes of different stemness subtypes (Figure 4E). For instance, TP53 was the most frequently mutated gene in both subtypes, but the mutation frequency in stemness subtype II was 13% higher than that in stemness subtype I. CSMD3 and PCL10 are unique high-frequency mutated genes in stemness subtype II, ranking the fifth and sixth; but stemness subtype I was characterized by high mutation frequency of ALB and RYR2. The above findings suggested that genomics differences between two stemness subtypes might lead to different statuses in immune infiltration and response to immunotherapy.

### Different stemness subtypes possessed different TIME statuses, Immunogenomics features and sensitivity to targeted therapy

**Figure 5:**
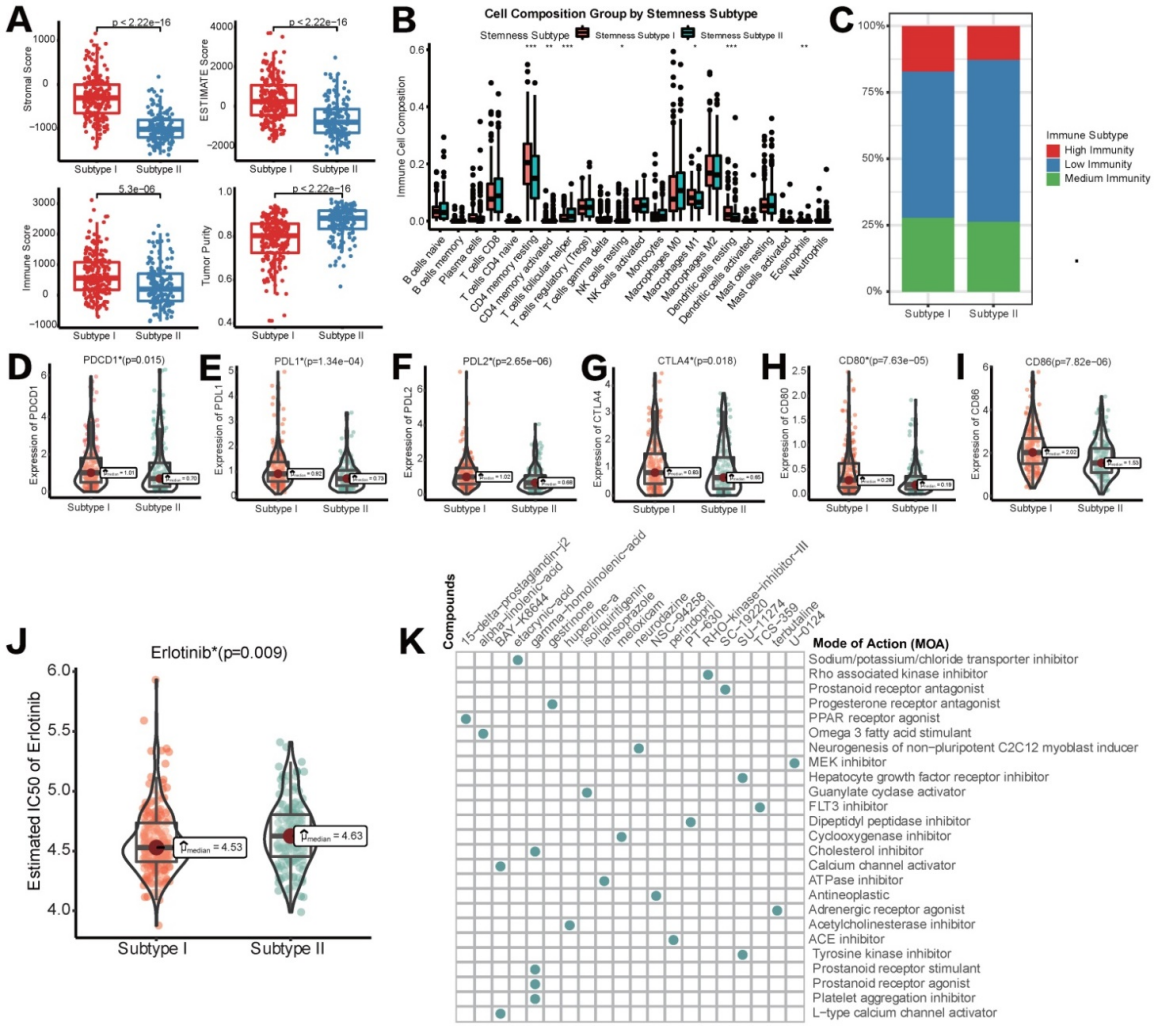
** Different TIME status, immunogenomic patterns and sensitivity to targeted therapy between two stemness subtypes. (A)** Comparisons of stromal score, immune score, ESTIMATE score and tumor purity between Stemness Subtype I and II. **(B)** Comparisons of the abundances of 22 immune cells in two subtypes by CIBERSORT. **(C)** Different proportions of immunity subgroups in two stemness subtypes. **(D-I)** Expression status of PD-1, PD-L1, PD-L2, CTLA-4, CD80 and CD86 in two stemness subtypes. **(J)** Different sensitivity responding to Erlotinib between two stemness subtypes illustrated by IC50. **(K)** cMAP analysis showed potential agents that might targeted 100 DEGs and their related MoA pathways. (*p < 0.05; **p < 0.01; ***p < 0.001; ****p < 0.0001; ns, *p* > 0.05)

As previously reported, we utilized ESTIMATE and CIBERSORT algorithms to elucidate different infiltration of TIME in two different stemness subtypes. As shown in Figure 5A, stromal score, immune score, ESTIMATE score and tumor purity all appeared to be lower in stemness subtype II compared with stemness subtype I, indicating the immunosuppressive microenvironment and low abundance of stromal cells in stemness subtype II. CIBERSORT to illustrate the infiltration abundances of immune cells also indicate that immune infiltration in stemness subtype II was partially suppressed than that in stemness subtype I (Figure 5B). Regarding the consistency between Stemness Subtypes and the aforementioned immunity subgroups, we observed that stemness subtype I contained most of the high-immunity HCCs, while Stemness Subtype II included a majority of low-immunity HCCs (Figure 5C). Furthermore, the expression status of common immune checkpoint genes was also determined in stemness subtypes I and II. As shown in Figure 5D-5I, both PD1/PD-L1/PD-L2 family and CTLA-4/CD80/CD86 family were higher expressed in stemness subtype I than subtype II, suggesting that patients in stemness subtype I might exert a better response to ICB administration.

**Figure 6:**
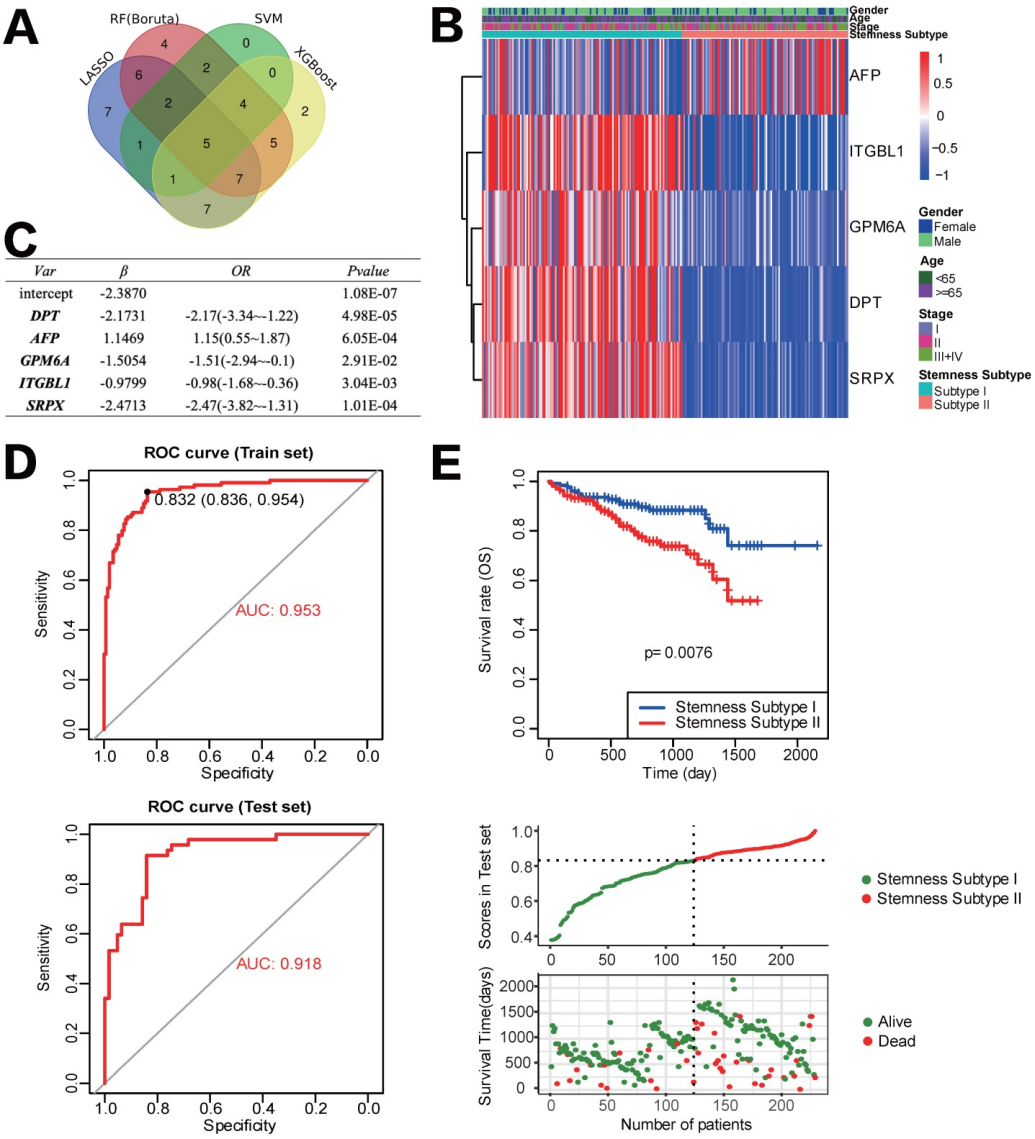
** Construction and validation of the Stemness Subtype classifier based on hub genes by machine learning methods. (A)** Venn diagram identified five hub genes that were shared by four feature selection algorithms. **(B)** Expression heatmap of five hub genes and integrated results of stemness subtypes and clinical features. **(C)** Results and parameters of multivariate logistic regression analysis that provided the calculating formula of Stemness Subtype classifier. **(D)** ROC curves of the Stemness Subtype Classifier in distinguishing two subtypes in the train set (Up) and test set (Down). **(E)** External verification of patients' overall survival for Stemness Subtype Classifier in HCC patients from ICGC datasets.

Subsequently, we tried to explore if different stemness subtypes had discriminating sensitivity to targeted therapy drugs or other potential compounds. As mentioned above, Lenvatinib was the first-line agent approved as a strategy of targeted therapy for HCC. Regrettably, in pRRophetic algorithm based on the GDSC database, data of Lenvatinib was not available. Therefore, we switched to Erlotinib as an alternative targeted therapeutic strategy. Mechanistically, both Erlotinib and Lenvatinib were classic receptor tyrosine kinase (RTK) inhibitors 30. As an EGFR inhibitor, Erlotinib was also reported to block the downstream secretion of VEGF, exerting synergistic anti-tumor effects with Lenvatinib, a VEGFR inhibitor 31. Figure 5J showed that IC50 of Erlotinib in HCC patients was significantly lower in stemness subtype I compared with subtype II, indicating a higher sensitivity to Erlotinib, and potentially Lenvatinib in patients with stemness subtype I. Then, CMap database was used to identify new potential compounds targeting the included 100 mRNAsi phenotype-based DEGs. 19 compounds enriched in 25 signaling pathways were identified (Figure 6H). These agents and their related pathways might synergistically promote immunotherapy targeting stemness features in HCC.

### Development and verification of the Stemness Subtype Classifier based on multiple machine learning algorithms

After removing the attributes with an absolute correlation of 0.75 or higher, 56 of 100 mRNAsi phenotype-based DEGs were left. We aimed to find core stemness subtype relevant features based on 56 mRNAsi-related DEGs to build a clinically applicable classifier that could conveniently predict the stemness subtype of HCC patients. For protecting against overfitting and eliminating noise in data, 365 HCC patients in the TCGA cohort were stratified into training (n=255) and testing (n=110) set at an equal ratio of 7:3, we applied 4 machine learning algorithms based on the training set. As shown in the Venn diagram in Figure 6A, a total of 36, 15, 35 and 31 mRNAsi phenotype-based DEGs were selected by LASSO, SVM-RFE, RF and XGBoost, separately; results of each algorithm were shown in Figure S2. After the intersection, a total of 5 hub DEGs that were converged by the results of four machine learning methods were identified, which were DPT, AFP, GPM6A, ITGBL1 and SRPX (Figure 6B). Then, multivariate logistic regression analysis was implemented to establish the diagnostic model. The computational equation of the Stemness Subtype Classifier was: -2.3870-2.1731*(expression of DPT) + 1.1469*(expression of AFP) - 1.5054*(expression of GPM6A) - 0.9799*(expression of ITGBL1) - 2.4713*(expression of SRPX). The optimal threshold for classification was 0.8322107, which meant that if a patient with a score > 0.8322107, he or she would be classified into Stemness Subtype I, else subtype II (Figure 6C). ROC curve proved that this classifier based on logistic regression was very reliable, with an AUC of 0.953 in the training set and an AUC of 0.918 in the internal testing set (Figure 6D).

For further validation of the Stemness Subtype Classifier, ICGC-LIHC dataset, a new independent cohort of HCC patients was enrolled. We calculated the score again for each patient based on the 5 hub genes and the same classifier formula and then divided patients into two subgroups based on the same optimal threshold of the classifier. ICGC cohort consisted of 126 patients with Stemness Subtype I (54.54%) and 105 patients with Stemness Subtype II (45.45%) (Figure 6E). K-M curve validated that patients in Stemness Subtype II had a worse OS compared with those in Subtype I, which is consistent with the results in the TCGA cohort, suggesting that this classifier was an independent prognostic factor in HCC.

## Discussion

The past decades have witnessed the rapid development of HCC stem cell related theory. Liver cancer stem cell (LIHC) subpopulations, just like CSCs in other tumors, are equipped with classic surface markers such as EpCAM, CD133, CD44, CD13, CD90, etc. In addition, agents targeting molecules in CSC pathways, like OMP-18R5, OMP-54F28 (Wnt/β-catenin signaling inhibitor), and LY2157299 (TGF-β inhibitor) are progressing to clinical trials for the supplement of HCC therapeutic strategies^32^. Current research on LIHC is guided into two directions. On the one hand, new biomarkers and CSC-based therapeutic strategies are still being proposed. For example, Wang X et al^33^ identified a liver progenitor specific gene RALYL that could promote HCC stemness *in vitro*; they confirmed that this developmental gene could upregulate TGF-β2 expression in an N6-methyladenosine (m6A) dependent manner. In the series studies published by Xu x et al^34^, ^35^, a ubiquitin-related gene named USP22 was proved as a glycolysis inducer by deubiquitinating and stabilizing HIF1α, which promoted hypoxia-induced HCC stemness; moreover, they constructed lipopolyplex nanoplatforms to deliver both USP22 shRNA and sorafenib, effectively inhibiting the development of HCC *in vitro* and *in vivo*. On the other hand, bioinformatics scientists and computational biologists have started to use existing CSC markers to calculate comprehensive stemness indexes through algorithms. In this study, we applied the OCLR machine-learning algorithm proposed by Malta TM and his colleagues. Combined with the PCBC dataset uploaded by Daily K^36^ and Salomonis N^37^, we calculated mRNAsi score for each patient, identified two distinct molecular clusters of stemness and further evaluated the differences between the two clusters in clinicopathological factors, prognosis and immune infiltration for HCC patients for the first time. This workflow has been confirmed stable and innovative to identify undiscovered biological mechanisms associated with the dedifferentiated oncogenic state. Hao L et al. 38 not only used the model to identify stemness features in medulloblastoma but also extended mRNAsi score to epigenetics, utilizing DNA methylation sequencing data to calculate the mDNAsi score and achieved good predictive efficiency. In our opinion, this computational method effectively compensates for the limitations of current CSC experimental research, such as low sorting efficiency and specificity, and a lack of phenotypically stable cell models *in vitro*. The application of computational biology in CSC research may have broad development prospects in the future.

The development of immunotherapy in HCC has been more optimistic than in many other malignancies. However, a lack of biomarkers that could predict the patient's response or assist in enhancing the efficacy of immunotherapy remains the largest obstacle to the further development of immunotherapy in HCC. CSCs, representing the core cell subgroups causing tumorigenesis, metastasis recurrence, and therapeutic resistance, might become the linchpin to improve the effectiveness of immunotherapy in HCC patients. In recent years, plenty of studies have identified complex signal crosstalk between immune cells and stromal cells in TME and CSCs of HCC. For example, Song M and his colleagues 39 reported a crosstalk signaling based on chemokine in HCC; they discovered that cancer-associated fibroblasts (CAFs) derived CLCF1 could increase the secretion of CXCL6 and TGF-β1 in tumor cells, enhancing cancer stemness; CXCL6 and TGF-β1, in turn, activated ERK 1/2 signaling of CAFs to create more CLCF1, forming a positive feedback loop to promote HCC progression. Wei Y et al. 40 discovered that IL-17A expressed and secreted by lymphatic endothelial cells in the TME could preferentially interact with CD133+ HCC cells *in vitro*, leading to the self-renewal and tumorigenesis by hepatoma stem cells; besides, CD133+ CSCs could, in turn, activate cytokine IL-17A expression in lymphatic endothelial cells. Similarly, Zhou S 41 proved that tumor-associated neutrophils could secret BMP-2 and TGF-β2, which triggered the expression of miR-301b-3p through paracrine and further induced the activation of NF-κB signaling, finally increasing stem cell characteristics in HCC cells *in vitro*. As feedback, these HCC cells with enhanced stemness could highly express the chemokine CXCL5 and recruit more neutrophils to infiltrate TME. Our study also confirmed the view that CSCs and TME are closely related. In the two classifiers we built in this study, which were mRNAsi score model (high vs. low subgroups) and stemness subtype model (Stemness Subtype I vs. Stemness Subtype II), patients with more obvious stemness characteristics or in the subgroup of poor prognosis tended to manifest as immunosuppression, increased expression of immunosuppressive checkpoints and decreased stromal scores, indicating that these models possessed the value of follow-up mechanism research and clinical application. Our study could potentially predict the status of tumor immune response and the response to immunotherapy in HCC patients.

To construct a clinically applicable predictor for stemness subtype, we screened the hub genes in 100 mRNAsi phenotype-based DEGs by four machine learning methods with different algorithm principles, which were LASSO, XGboost, SVM-RFE and RFB. In the field of computer science, these four algorithms had different effects in solving different situations. When solving medical issues, it was difficult for us to confirm which method could achieve the best accuracy and precision. Huang J et al. 42 used to compare multiple machine learning algorithms in medical data. They found that in the scenario of predicting the relationship between intradialytic hypotension and patients' mortality, both RFB and XGboost seemed to be superior in predictive performance. Otherwise, the integrated machine learning based model tended to generate meaningful data for the prediction of the clinical revolution of cancers 43, 44.

Herein this study, we set the most stringent selection criteria, which was to take the intersection of four results, to make sure that we obtained hub genes that could best represent every stemness subtype. The 5 selected genes were DPT, AFP, GPM6A, ITGBL1 and SRPX. Literature review indicated that all these five genes could be expressed and/or secreted by cancer parenchymal cells and were extracellular, cell adhesion and/or immune response related. DPT represents the gene of an extracellular stromal protein Dermatopontin, which is critical in cell-matrix interactions and activation of TGF-β signaling. In HCC, DPT was regarded as a proliferation inhibitor and metastasis promoter by regulating chemokine CXXC4, which in turn targeted Wnt signaling to further inhibit HCC cell growth but promote metastasis 45. AFP is a major plasma protein produced by the liver; it has been widely accepted that AFP was highly expressed in HCC cells and elevated AFP protein level in plasma has been applied clinically as an early diagnostic and prognostic predictor in HCC patients for decades 46. GPM6A is related to mesoderm differentiation during development, including differentiation and migration of neuronal stem cells 47; SRPX is an X chromosome located gene involved in cell adhesion and cell membrane remodeling 48; however, the role of these two genes in HCC was largely unknown. ITGBL1 is a secreted protein of the EGF-like protein family. It participates in the invasion, migration and immune escape of several cancers. Huang W and his colleagues 49 proved that ITGBL1 was highly expressed in HCC patients with a prognostic value. Mechanically, it activated TGF-β/Smads signaling and further regulated EMT-related genes *in vitro*. Thus, ITGBL1 might be a key regulator linking stemness and immune response in cancers.

As for other diagnostic models in HCC, Feng, et al. 50 identified 17 immune-related gene pairs to explore its responsiveness to ICB and targeted therapy via the LASSO-Cox algorithm. Xu et al.^51^ constructed a 5-gene (ATG10, IL18RAP, PRKCD, SLC11A1 and SPP1) risk model through univariate Cox and LASSO analyses for prognostic evaluation. Not only the value for prediction of these models was less powerful than our classifier based on multiple machine learning algorithms, but the clinical utility of our classifier was also stronger and better. Furthermore, we were the first to integrate LASSO, SVM-RFE, RF and XGBoost to construct our novel diagnostic classifier in HCC. However, the limitation of our studies are as follows, since HCC patients who received neoadjuvant immunotherapy are limited, the association between the classifier and immunotherapy should be further confirmed based on the immunotherapy cohort in our further research. Otherwise, although we validated the predictive efficiency in the TCGA testing set and ICGC cohort, more experimental data *in vivo* and vitro need to be replenished.

## Conclusion

To sum up, our study provided more evidence that CSCs played a pivotal role in the regulation of immune infiltration status in the tumor microenvironment and immunotherapeutic response in HCC patients. We constructed an innovative and clinically feasible HCC stemness subtype classifier that could become both a guide for further mechanism research between CSCs and TME and a potential approach for selecting the immunotherapy susceptible responders in the future.

## Supplementary Material

Supplementary figures.

## Abbreviations

AUC: area under the ROC curve
CAF: cancer-associated fibroblast
CCLE: Cancer Cell Line Encyclopedia
CDF: cumulative distribution function
CM: consensus matrix
CMap: Connectivity Map
CNV: copy number variation
CSC: cancer stem cell
DEG: differentially expressed gene
EMT: epithelial-mesenchymal transition
ESTIMATE: Estimation of STromal and Immune cells in MAlignant Tumor tissues using Expression data
FACS: fluorescence-activated cell sorting
FC: fold change
GO: Gene Ontology
GSVA: Gene set variation analysis
HCC: hepatocellular carcinoma
ICB: immune-checkpoint blockade
ICGC: International Cancer Genome Consortium
ICI: immune checkpoint inhibitor
IC50: half-maximal inhibitory concentration
KEGG: Kyoto encyclopedia of genes and genomes
K-M: Kaplan-Meier
LASSO: least absolute shrinkage and selection operator
MACS: magnetic-activated cell sorting
MDSC: myeloid-derived suppressor cell
MoA: mode of action
mRNAsi: stemness index
OCLR: one-class logistic regression
OS: overall survival
PCBC: Progenitor Cell Biology Consortium
PSC: pluripotent stem cell
RFB: Random Forest and Boruta
ROC: receiver operating characteristic
ssGSEA: single sample Gene Set Enrichment Analysis
SVM-RFE: support vector machine recursive feature elimination
TACE: transarterial chemoembolization
TAM: tumor-associated macrophages
TCGA: The Cancer Genome Atlas
TIME: tumor immune microenvironment
TMB: tumor mutation burden
TIL: tumor-infiltrating lymphocyte
XGBoost: extreme gradient boosting
