# Supplementary figures and images for: Integrated Machine Learning and Bioinformatic Analyses Constructed a Novel Stemness-Related Classifier to Predict Prognosis and Immunotherapy Responses for Hepatocellular Carcinoma Patients
Source: Int J Biol Sci. 2022 Jan 1;18(1):360–73. doi: 10.7150/ijbs.66913 (PMC8692161; doi:10.7150/ijbs.66913)

Figure S1

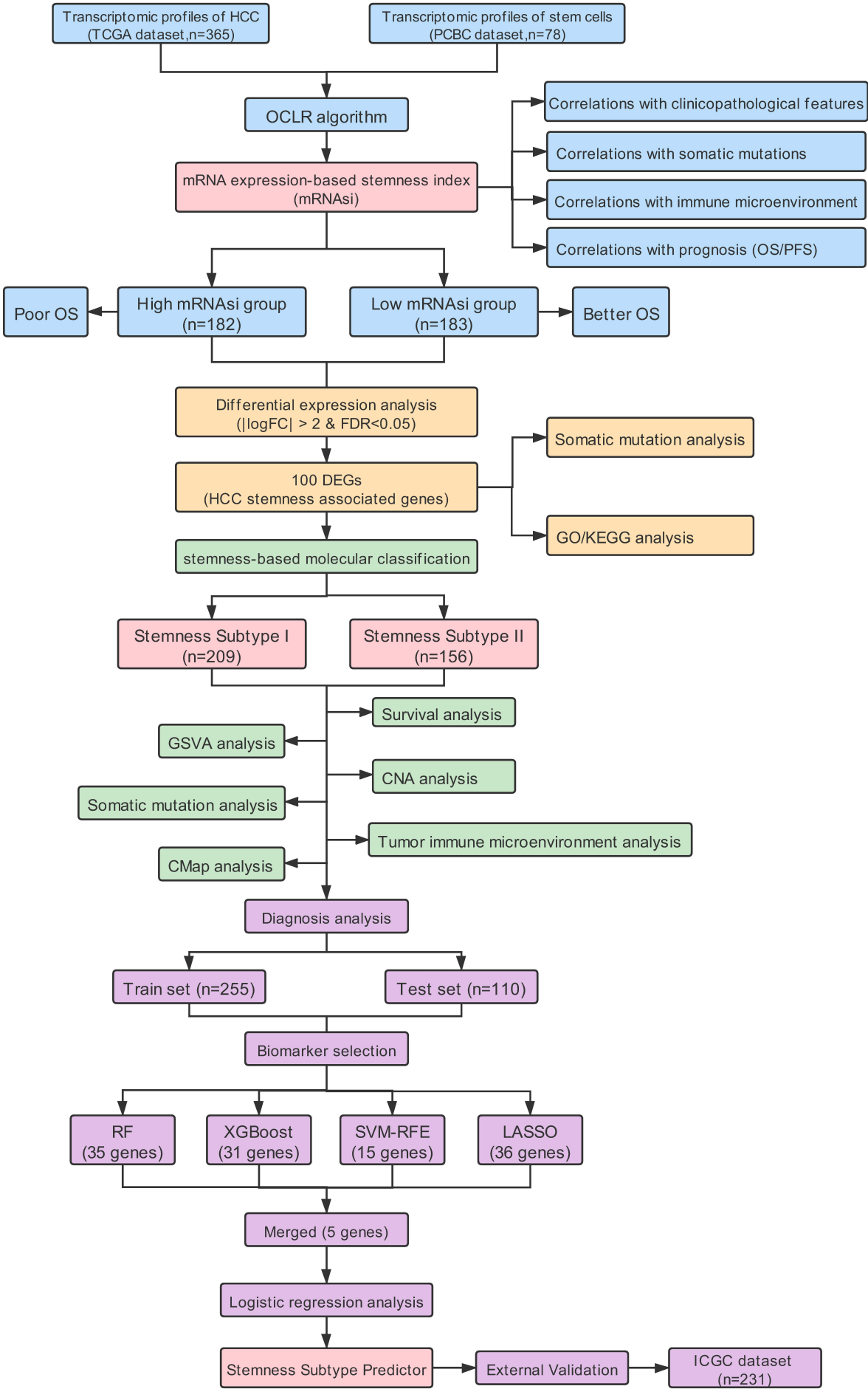

Figure S2

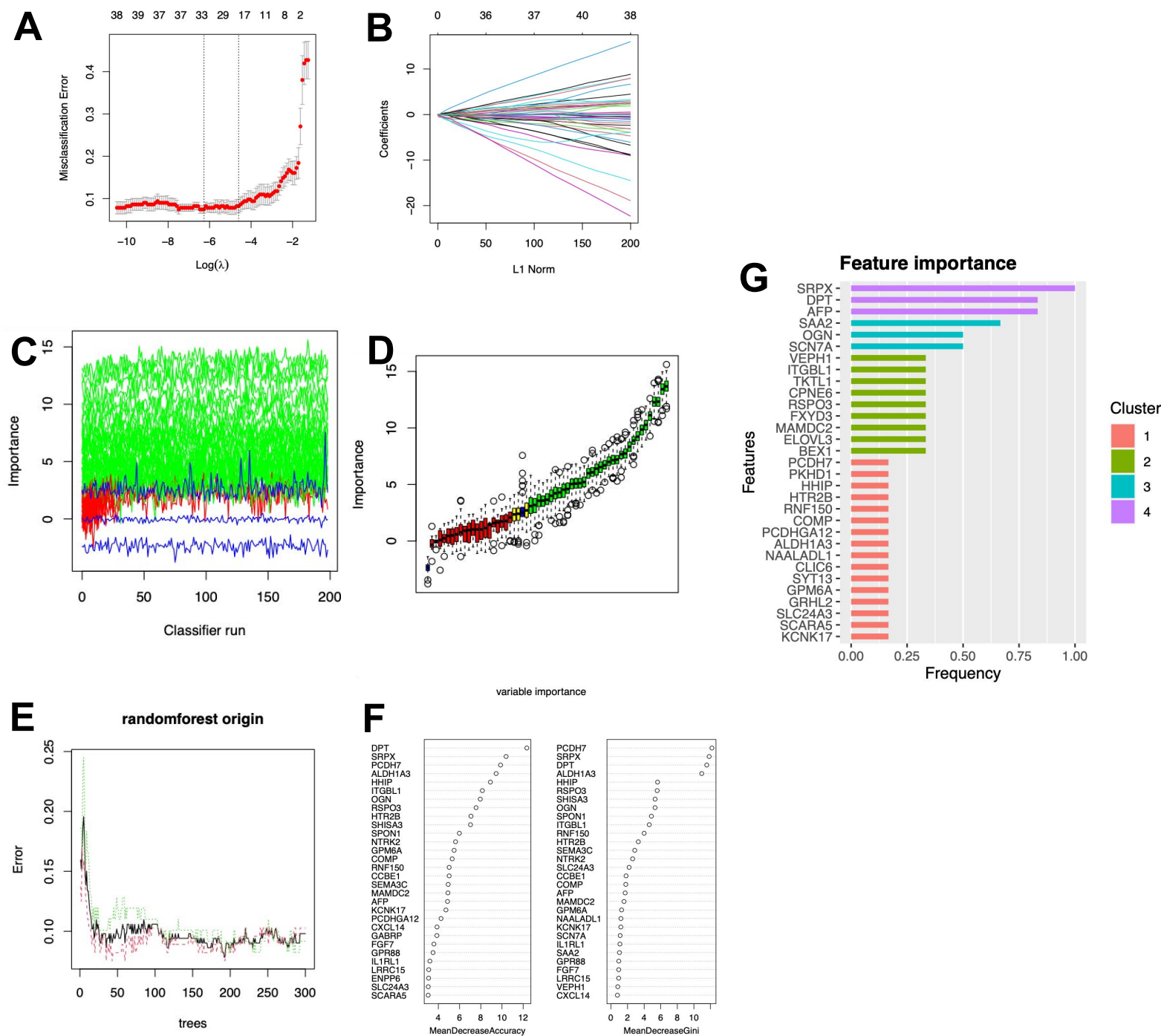

Supplement: Supplementary file 1 — Supplementary figures. [file ijbsv18p0360s1.pdf]
